# Metal-Dependent Regulation of *ATP7A* and *ATP7B* in Fibroblast Cultures

**DOI:** 10.3389/fnmol.2016.00068

**Published:** 2016-08-18

**Authors:** Malgorzata Lenartowicz, Torben Moos, Mateusz Ogórek, Thomas G. Jensen, Lisbeth B. Møller

**Affiliations:** ^1^Department of Genetics and Evolution, Institute of Zoology, Jagiellonian UniversityKrakow, Poland; ^2^Section of Neurobiology, Biomedicine, Institute of Medicine and Health Technology, Aalborg UniversityAalborg, Denmark; ^3^Department of Biomedicine, Aarhus UniversityAarhus, Denmark; ^4^Applied Human Molecular Genetics, Kennedy Center, Department of Clinical Genetics, Copenhagen University Hospital – RigshospitaletGlostrup, Denmark; ^5^Department of Science, Systems and Models, Roskilde UniversityRoskilde, Denmark

**Keywords:** copper, menkes disease, wilson disease, regulation, iron–copper interaction

## Abstract

Deficiency of one of the copper transporters ATP7A and ATP7B leads to the rare X-linked disorder Menkes Disease (MD) or the rare autosomal disorder Wilson disease (WD), respectively. In order to investigate whether the *ATP7A* and the *ATP7B* genes may be transcriptionally regulated, we measured the expression level of the two genes at various concentrations of iron, copper, and insulin. Treating fibroblasts from controls or from individuals with MD or WD for 3 and 10 days with iron chelators revealed that iron deficiency led to increased transcript levels of both *ATP7A* and *ATP7B*. Copper deficiency obtained by treatment with the copper chelator led to a downregulation of *ATP7A* in the control fibroblasts, but surprisingly not in the WD fibroblasts. In contrast, the addition of copper led to an increased expression of *ATP7A*, but a decreased expression of *ATP7B*. Thus, whereas similar regulation patterns for the two genes were observed in response to iron deficiency, different responses were observed after changes in the access to copper. Mosaic fibroblast cultures from female carriers of MD treated with copper or copper chelator for 6–8 weeks led to clonal selection. Cells that express the normal *ATP7A* allele had a selective growth advantage at high copper concentrations, whereas more surprisingly, cells that express the mutant *ATP7A* allele had a selective growth advantage at low copper concentrations. Thus, although the transcription of *ATP7A* is regulated by copper, clonal growth selection in mosaic cell cultures is affected by the level of copper. Female carriers of MD are rarely affected probably due to a skewed inactivation of the X-chromosome bearing the *ATP7A* mutation.

## Introduction

ATP7A and ATB7B are copper ATPases of the P-type 1B family. Mutations in the genes that encode these copper transporters are responsible for Menkes disease (MD) and Wilson disease (WD), respectively. MD is a severe X-linked copper deficiency disorder which typically leads to death before the age of 3 years. The copper deficiency is caused by a defective ATP7A-mediated transport of copper from the intestine into the portal circulation. As a consequence, the clinical features of MD are a malfunction of a large number of copper-requiring enzymes. WD is an autosomal copper toxicity disorder. Typically, copper accumulates in the liver as a result of a disrupted ATP7B-driven copper export from the hepatocytes. Both transporters are responsible for copper export, but in different cell types. The *ATP7A* is expressed in almost all cell types and tissues, but its expression levels differ among the cells and tissues and are age-dependent (Lenartowicz et al., 2010). In contrast, *ATP7B* is predominantly expressed in the adult liver Lenartowicz et al., 2015).

Fibroblasts from MD patients accumulate large amounts of copper as a result of impaired copper extrusion in contrast to fibroblasts from WD patients, in which no accumulation of copper is observed (own unpublished results). Both *ATP7A* and *ATP7B* are expressed in fibroblasts, but the expression level of *ATP7A* is approximately 10 times higher than that of *ATP7B* (own unpublished results).

Female carriers are mosaics of wild-type and mutant cells due to random X-inactivation, and are rarely affected. A likely explanation is that the majority (more than 90%) of the females have a skewed inactivation of the X-chromosome bearing the *ATP7A* mutation and therefore, predominantly express the normal allele (Møller et al., 2012). However, from a large number of fibroblast cultures we were able to identify cultures from three MD carriers, including one manifesting carrier with MD, with an even or almost even X-inactivation pattern (Møller et al., 2012).

Not much is known about the possible transcriptional regulation of *ATP7A* and *ATP7B*. It seems that regulation and copper homeostasis occur predominantly in post-translational mechanisms (van den Berghe and Klomp, 2010). This study was designed to test the transcriptional effect on *ATP7A* and *ATP7B* in fibroblast cultures after short (3 days) or longer (10 days) term treatment with iron and copper and chelators thereof, and insulin. Short-term treatment might exhibit a rapid effect on transcriptional levels, whereas long-time treatment may also involve cellular selection. We treated fibroblasts obtained from controls with both intact *ATP7A* and intact *ATP7B*, fibroblasts from MD with only intact *ATP7B*, and fibroblasts from WD with only intact *ATP7A*.

Furthermore, we looked at clonal growth selection as an effect of functional *ATP7A* gene expression, and the cellular concentration of copper. Mosaic cultures obtained from female MD carriers with an even X-inactivation pattern, were treated with copper or the copper chelator (Bathocuproine disulphonate, BCS).

## Results

### Transcriptional Expression of *ATP7A* and *ATP7B* in Fibroblast Cultures

To test for a possible transcriptional regulation of *ATP7A* and *ATP7B*, we treated fibroblasts from patients with MD or WD and control fibroblasts with various chemicals and hormones: BCS (copper chelator, Bathocuproinedisulfonic acid disodium salt), DEDTC (copper chelator, Sodium diethyldithiocarbamate trihydrate), CuCl2, Ferrozine (Fe2+ chelator) DTPA (Diethylenetriaminepentaacetic acid, Fe2+ chelator) and insulin. The cells were treated for 3 days and 10 days before RNA was harvested and analyzed using RT-PCR. The expression levels of *ATP7A* and *ATP7B* were calculated and normalized to the expression level of the housekeeping genes GAPDH (**Table 1**).

**Table 1A:** *ATP7A* expression in control, MD and WD cells after 3 and 10 days of culture.

	*ATP7A/GAPDH* EXPRESSION					
	Control Fibroblasts		MD Fibroblasts		WD fibroblasts	
	After 3 days	After 10 days	After 3 days	After 10 days	After 3 days	After 10 days
Standard medium	0.88 ± 0.17 (12)	1.49 ± 0.49 (13)	0	0	0.99 ± 0.01 (5)	1.51 ± 0.08 (5)
BCS	0.98 ± 0.46 (12)	0.87 ± 0.39** (12)	0	0	1.14 ± 0.08 (5)	1.66 ± 0.09 (5)
DEDTC	1.18 ± 0.48 (4)	1.22 ± 0.26 (5)	0	0	1.06 ± 0.13 (5)	1.33 ± 0.00 (5)
CuCl_2_	1.39 ± 0.28** (8)	1.25 ± 0.27 (12)	0	0	1.30 ± 0.15** (5)	1.81 ± 0.13** (5)
Ferrozine	1.38 ± 0.34** (12)	1.54 ± 0.53 (13)	0	0	1.44 ± 0.17** (5)	1.70 ± 0.17* (5)
DTPA	1.39 ± 0.38* (5)	1.54 ± 0.58 (5)	0	0	1.19 ± 0.07** (5)	1.91 ± 0.29* (5)
FeCl_3_	0.78 ± 0.32 (12)	1.44 ± 0.36 (13)	0	0	0.97 ± 0.06 (5)	1.92 ± 0.43 (5)
Insulin	1.11 ± 0.19* (10)	1.11 ± 0.66 (9)	0	0	1.06 ± 0.11 (5)	1.67 ± 0.19 (5)
Insulin+ BCS	1.43 ± 0.64 (6)	0.82 ± 0.23*** (9)	0	0	1.01 ± 0.3 (5)	1.44 ± 0.17 (5)
Insulin + CuCl_2_	1.20 ± 0.27* (11)	1.42 ± 0.60 (13)	0	0	1.17 ± 0.16 (5)	1.64 ± 0.07* (5)

*^∗^significantly different from standard medium *P* < 0.05. ^∗∗^significantly different from standard medium *P* < 0.01. ^∗∗∗^significantly different from standard medium *P* < 0.001.Number of samples is in brackets.*

**Table 1B:** *ATP7B* expression in control, MD and WD cells after 3 and 10 days of culture.

	*ATP7B/GAPDH* EXPRESSION					
	Control fibroblasts		MD fibroblasts		WD fibroblasts	
	After 3 days	After 10 days	After 3 days	After 10 days	After 3 days	After 10 days
Standard medium	1.06 ± 0.15 (11)	1.24 ± 0.43 (12)	2.41 ± 0.13 (2)	1.93 ± 0.05 (2)	0.69 ± 0.08 (5)	1.58 ± 0.19 (5)
BCS	1.18 ± 0.28 (12)	1.30 ± 0.46 (13)	Lack of data	1.92 ± 0.04 (2)	0.80 ± 0.23 (5)	1.27 ± 0.23 (5)
DEDTC	1.01 ± 0.19 (4)	1.10 ± 0.45 (5)	1.84 ± 0.05 (2)	1.84 ± 0.05 (2)	0.65 ± 0.12 (5)	0.96 ± 0.02 (2)
CuCl_2_	1.05 ± 0.32 (8)	0.89 ± 0.28* (6)	2.27 ± 0.05 (2)	1.16 ± 0.02 (2)	0.81 ± 0.22 (5)	1.10 ± 0.29* (5)
Ferrozine	2.03 ± 0.91** (12)	1.92 ± 0.44** (11)	4.83 ± 0.13 (2)	1.80 ± 0.00 (2)	1.20 ± 0.24* (5)	1.44 ± 0.27 (5)
DTPA	1.20 ± 0.22 (6)	1.75 ± 0.45* (6)	2.40 ± 0.19 (2)	2.58 ± 0.17 (2)	0.77 ± 0.13 (5)	1.61 ± 0.20 (5)
FeCl_3_	1.09 ± 0.22 (12)	1.63 ± 0.31* (11)	Lack of data	1.73 ± 0.03 (2)	0.78 ± 0.23 (4)	1.23 ± 0.33 (5)
Insulin	1.11 ± 0.17 (12)	1.05 ± 0.33 (11)	2.57 ± 0.42 (2)	1.64 ± 0.02 (2)	0.93 ± 0.21 (5)	1.50 ± 0.33 (5)
Insulin + BCS	1.03 ± 0.28 (5)	1.25 ± 0.38 (9)	1.19 ± 0.01 (2)	0.71 ± 0.02 (2)	0.48 ± 0.18 (5)	1.43 ± 0.13 (5)
Insulin+ CuCl_2_	1.36 ± 0.51 (9)	1.19 ± 0.49 (13)	2.36 ± 0.03 (2)	1.36 ± 0.04 (2)	0.83 ± 0.33 (5)	1.26 ± 0.38 (5)

*^∗^significantly different from standard medium *P* < 0.05. ^∗∗^significantly different from standard medium *P* < 0.01. Number of samples is in brackets.*

Treatment with iron chelator (Ferrozine, DTPA) led to an increased expression of both *ATP7A* and *ATP7B*. Treatment with FeCl3 led to an increased expression of *ATP7B* in the control fibroblasts, but only after 10 days. Treatment with insulin led to an increased expression of *ATP7A*, whereas no effect was observed for *ATP7B*. The effect was transient (only observed after 3 days) and was only significant in control cells. Furthermore, treatment with CuCl_2_ led to an increased expression of *ATP7A*, but a decreased expression of *ATP7B*. The effect on *ATP7A* was only transient in control cells (3 days only), whereas it was stable in WD cells (3 and 10 days). This indicates that in WD cells with no functional *ATP7B* gene, an increased expression of *ATP7A* is of significant importance in the presence of copper overload. In contrast, a decreased expression of *ATP7B* was only observed after 10 days of CuCl2 treatment. Interestingly, treatment with copper chelator (BCS) led to a decreased expression of *ATP7A*, but only in control cells and only after 10 days of treatment. No decrease of *ATP7A* was observed in WD cells. BCS had no effect on *ATP7B*.

*ATP7A* transcripts could not be detected in MD fibroblasts, because the *ATP7A* gene in these cells was almost completely deleted (ex2–ex23) There was some expression of *ATP7B* in WD fibroblasts, but since the mutation [c.2333G > T, p.(Arg778Leu)] might affect splicing and/or RNA stability its expression was relatively low. Interestingly, however, the expression level of *ATP7A* increased after long-term culture in the control medium, whereas the expression level of *ATP7B* was more stable except in the WD fibroblasts, where a significant increase was observed. Importantly, the expression of ATP7B was constantly high in MD fibroblasts.

Since *ATP7A* expression was increased in a high copper environment and decreased in a low copper environment, we tested the impact of *ATP7A* on cell survival as an effect of copper concentration. In order to perform this test, clonal growth selection was investigated in mosaic cultures from heterozygous females.

### Copper-Dependent Clonal Selection in Mosaic Cultures

Fibroblasts from MD carriers with even X-inactivation patterns are mosaic with respect to functional *ATP7A* due to the inactivation of one of the two X chromosomes in each cell. A fraction of the cells express only mutated *ATP7A* transcript and behave like cells from an MD patient with an increased copper uptake, whereas other cells express normal *ATP7A* transcript and behave like control cells. Such cultures are suited for determining the effects of functional versus mutated *ATP7A*, since the cells potentially only differ with respect to genes expressed from the two X-chromosomes.

The cells were maintained in normal medium (control conditions), medium with CuCl2, or medium with BCS for several weeks, respectively, before DNA was harvested and the X-inactivation pattern determined. The methylation status of the androgen receptor (AR) gene was used as an indicator for X-chromosome inactivation. DNA from the index male was included in the X-inactivation test to identify the pattern of AR on the mutated allele.

**FIGURE 1:**
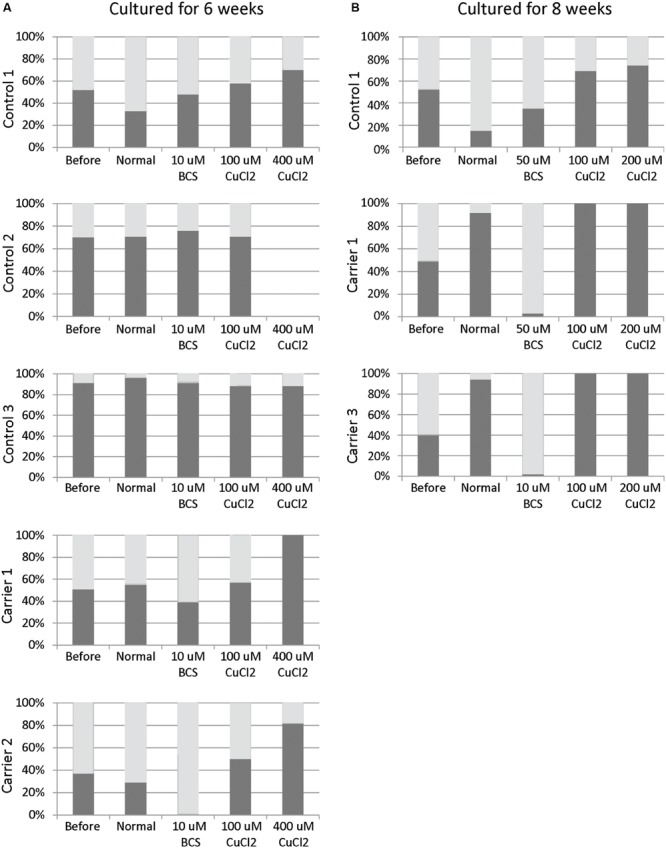
**X-inactivation patterns before and after 6 or 8 weeks of treatment with CuCl_2_ or BCS.** Fibroblasts were obtained from female carriers of *ATP7A* mutations or from females with no mutations in the *ATP7A* gene (control samples). The X-inactivation patterns were measured before and after 6 **(A)** or 8 **(B)** weeks of culture using normal medium or normal medium with different concentrations of BCS or CuCl_2_ as indicated in the figure. Each column shows the inactivation percentage of the two X-chromosomes (gray and black bars, respectively). In the carriers, the black bar represents inactivation of the X-chromosomes with *ATP7A* mutations, whereas the gray bar represents inactivation of the X-chromosomes without *ATP7A* mutations. Control 1 (3540-86H); Control 2: (M95-24668H); Control 3: (M85-2427H); Carrier 1: (3373-84H, heterozygote for the *ATP7A* mutation p.Gly1315Arg); Carrier 2: (3372-84H, heterozygote for the *ATP7A* mutation p.Gly1315Arg); Carrier 3: (M85-3426H (F1), heterozygote for the *ATP7A* mutation c.1946+5G > A).

Skewed inactivation of cells that express the mutant X-chromosome was observed after cultivation in the presence of 400 μM CuCl_2_ (ratio 100:0 in carrier 1 and 82:18 in carrier 2), indicating a selection of cells with the X-chromosome harboring the normal *ATP7A* gene active. In contrast, selection for cells with the mutant X-chromosome active was observed after incubation with 10 μM BCS (ratio 0:100 in carrier 2 and 39:61 in carrier 1). No significant effects were observed in the normal control cells (**Figure 1A**).

**FIGURE 2:**
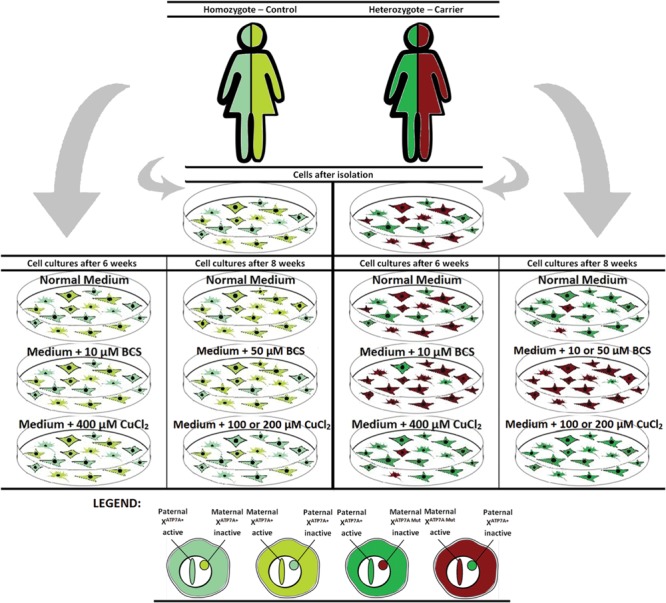
**Illustration of the outcome after copper or BCS treatment of mosaic fibroblast cultures for 6–8 weeks**.

To challenge the system further we repeated the experiment, but cultivated the heterozygous fibroblasts (carrier 1 and carrier 3) for 8 weeks instead of 6 weeks in the presence of either BCS (10 or 50 μM as indicated) or CuCl_2_ (100 and 200 μM). After 8 weeks, the cells’ ability to grow was almost exhausted. In the presence of BCS, almost all cells in the population had the mutant X chromosome active, whereas in the presence of CuCl_2,_ the entire cell population had the normal X-chromosome active. After 8 weeks, skewed increased X-inactivation of the X-chromosome harboring the mutated *ATP7A* allele was also observed in the cells grown in normal medium (**Figure 1B**). An illustration of the experiment and its outcome is shown in **Figure 2**.

### The Expression of *ATP7A* Transcript in Copper- and BCS-Treated Mosaic Cultures

**FIGURE 3:**
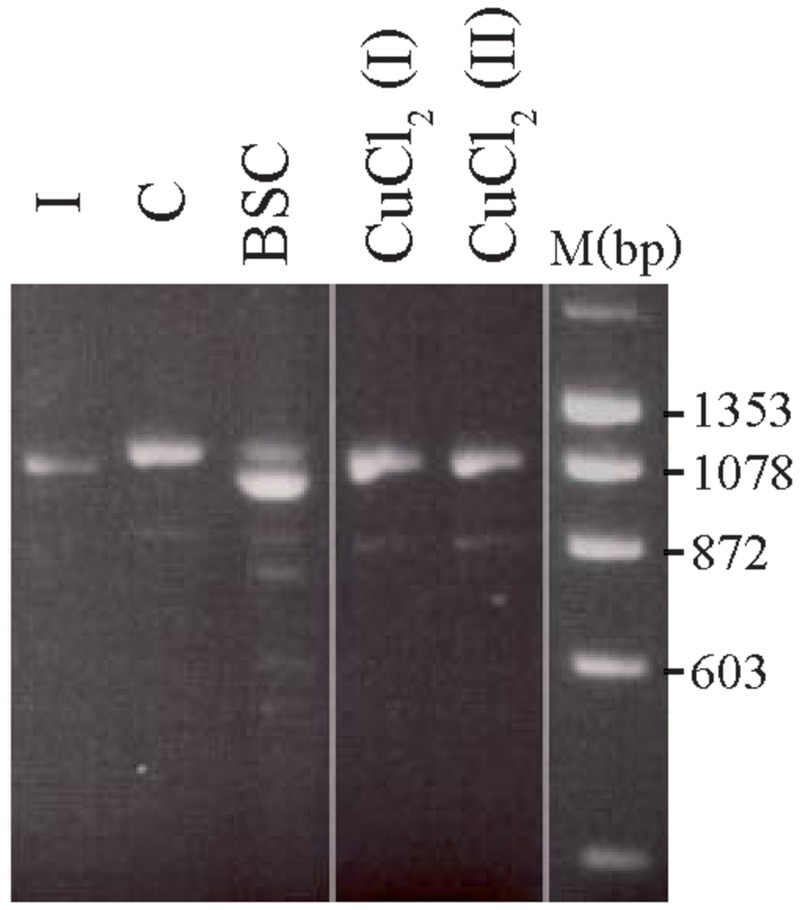
**Selection of the two different fibroblast cell types from carrier 3, heterozygous for the mutation c.1946+5G > A.** The fibroblasts were BCS and/or copper treated for 8 weeks. A cDNA fragment from exon 6 to exon 10 spanning the mutation was amplified by PCR with the primer-pair 5′-gccactgtgatagaaaatgctgat-3/′5′-caatagttgcttctgtagcttgtagtg-3′. I: cDNA from a male index with the mutation c.1946+5G > A, expressed only a mutant transcript in which exon 8 is missing. C: cDNA from heterozygous fibroblasts cultured in normal medium that predominantly expressed the wild-type transcript. BCS: cDNA from heterozygous fibroblasts, cultured in the presence of BCS, predominantly expressed a product in which exon 8 is missing. CuCl_2_ (I) and CuCl_2_(II): cDNA from heterozygous fibroblasts cultured in the presence of 100 μM CuCl_2_ (I) or 200 μm CuCl_2_ (II), predominantly expressed the wild-type product with exon 8. The PCR products were separated on a 2% agarose gel and visualized with ethidium bromide.

The treated 8-week-old cultures from carrier 3 were further used to investigate whether the X-inactivation pattern, hence reflecting the expression of mutant versus normal *ATP7A* transcripts using RT-PCR analysis (**Figure 3**). Carrier 3 is heterozygous for the mutation IVS8+5G > A. This mutation leads to 100% skipping of exon 8 in the index male (**Figure 3**, lane I). The amount of expressed wild-type versus mutant transcript can therefore easily be visualized after RT-PCR. Analyzing RNA from the mosaic cell culture grown in normal medium as well as in copper-supplemented medium, resulted in a PCR product identical to the wild-type transcript, whereas the major transcript obtained from cells grown in the presence of BCS corresponded to a mutant transcript: a transcript without exon 8, identical to the transcript observed in the index male patient (**Figure 3**, lane I). However, a faint band corresponding to the wild-type transcript was also observed. These results indicate that the expression of *ATP7A* follows the X-inactivation pattern.

## Discussion

Both P-type ATPases (ATP7A and ATP7B), are involved in copper eﬄux and intracellular sequestration. The overall topologies and structures of ATP7A and ATP7B are similar and they are approximately 60% homologous. They have eight transmembrane domains with six copper-binding motifs in the cytoplasmic N-terminal region. The N-terminal metal-binding domains interact with ATOX1 and receive copper in the form of Cu^+^. The transmembrane hydrophobic domains (TMDs) anchor the protein in the membrane and form a channel for the ATP-dependent transport of copper ions across the plasma or intracellular membranes. Cellular ATP7A and ATP7B play a dual role depending on the copper status. Under normal physiological conditions they are TGN-resident proteins and mediate copper loading of copper-dependent enzymes in the secretory pathway. When the intracellular copper concentration is elevated, ATP7A and ATP7B – after their re-localization/re-distribution to the plasma membrane or to vesicles – are essential for copper eﬄux or secretion. ATP7A and ATP7B contain similar catalytic domains and functional motifs that govern their intracellular cycling between the TGN and the plasma membrane (La Fontaine and Mercer, 2007; Lutsenko et al., 2007; Gupta and Lutsenko, 2009; Barry et al., 2010).

However, whereas both ATP7A and ATP7B preserve cellular copper homeostasis by expelling copper ions from the cell’s interior to prevent toxic accumulation, there are some differences. When the cells are exposed to a rise in copper concentrations, ATP7A is translocated to the basolateral membrane in polarized cells, whereas ATP7B is transported to the apical membrane (Mercer et al., 2003; La Fontaine and Mercer, 2007; Gupta and Lutsenko, 2009; Veldhuis et al., 2009; Barry et al., 2010). Furthermore, whereas ATP7A in the secretory pathway, delivers copper to lysyl oxidase, tyrosinase, dopamine-β-hydroxylase, and peptidylglycine-α-amidating monooxygenase among others (Petris et al., 2000; Hardman et al., 2007a; Lutsenko et al., 2007), the ATP7B protein participates in copper-loading of apo-ceruloplasmin (Barry et al., 2010).

We found both similarities and differences in the transcriptional regulation of *ATP7A* and *ATP7B*. A summary is shown in **Table 2**. We found that treatment with insulin led to a transient increase in *ATP7A* transcript, but had no effect on *ATP7B* transcript in control fibroblasts. This observation is in agreement with previous observations by Hardman et al. (2007b) who reported that treating Jeg-3 cells with insulin for 72 h (3 days) leads to an increase in the *ATP7A* transcript but has no effect on the *ATP7B* transcript. Insulin (and ostrogen) also changed the localization of ATP7A in the Jeg-3 cells; ATP7A was more diffusely distributed in the cytoplasm with increased accumulation at the basolateral membrane relative to the untreated cells (Hardman et al., 2007a). Our results indicate that the effect of transcriptional regulation of the *ATP7A* gene by insulin is not restricted to the placental cells and is closely related to the control of copper transport from mother to fetus, but it is also observed in other cell types.

**Table 2:** Summary of *ATP7A* and *ATP7B* transcriptional regulation in fibroblast cultures.

	*ATP7A* (control cells)	*ATP7A* (WD cells)	*ATP7B* (control cells)	*ATP7B* (WD cells)	*ATP7B* (MD cells)
+ insulin	↑	No effect	No effect	No effect	No effect
- Fe	↑	↑	↑	↑	↑
+ Fe	No effect	No effect	↑	No effect	No effect
- Cu	↓	No effect	No effect	No effect	No effect
+ Cu	↑	↑	↓	↓	No effect

Iron deficiency led to an increased expression of both *ATP7A* and *ATP7B*, but copper-deficiency only led to a decreased expression of *ATP7A*. The addition of copper led to an increased expression of *ATP7A*, but a decreased expression of *ATP7B*. The changes in transcripts (increase or decrease) were less than twofold. We have previously shown that the relative transcriptional level of *ATP7A* in control fibroblasts vary by almost a factor 2 (Paulsen et al., 2006) making it unlikely that the observed transcriptional regulation has any physiological significance.

Interactions between iron and copper metabolism were documented over a century ago, but the homeostatic effects at the molecular level and the involvement of *ATP7A* and *ATP7B* in this regulation have not been fully elucidated. Interaction between iron and copper is essential for metal homeostasis because, e.g., copper-dependent ferroxidases like ceruloplasmin, hephaestin, and zyclopen facilitate dietary iron absorption by oxidizing ferrous Fe^++^ to ferric Fe^+++^ iron (Vulpe et al., 1999; Collins et al., 2010; Gambling et al., 2011; Vashchenko and MacGillivray, 2013). Any impairment to the incorporation process of copper results in the secretion of an apo-ceruloplasmin that lacks any ferroxidase activity, and is rapidly degraded in the plasma (Hellman and Gitlin, 2002; Kono, 2013). *Atp7b* -/- mice, which constitute an animal model of WD, display disturbances in iron metabolism related to low serum ceruloplasmin oxidase activity (Merle et al., 2010). Furthermore, a complex interplay between these two metals seems to involve multiple copper-dependent regulatory mechanisms that act in concert to control the expression of ferroportin, the only identified ferrous iron transporter that exports iron from cells to plasma (Xie and Collins, 2011; Starzyński et al., 2013).

An increased expression of *ATP7A* has previously been identified in intestinal epithelial cells of iron-deficient rats and confirmed by *in vivo* and *in vitro* analyses (Collins et al., 2005, 2009; Collins, 2006). During iron deficiency many tissues become hypoxic. Cellular responses to hypoxia are mainly regulated by the activation of transcription factors called hypoxia-inducible factors (HIFs) (Hashimoto and Shibasaki, 2015). Iron deficiency in the intestinal epithelial cells leads to the upregulation of the *ATP7A* gene and iron-homeostasis related genes: the cell surface ferrireductase gene *Dtcyb*, the iron ion transporter gene *DMT1* and the basolateral iron exporter ferroportin gene (*FPN*) (Xie and Collins, 2011, Xie and Collins, 2013). Results of previous studies indicate that HIF2α binds to the promotor region of the *ATP7A* gene, the analysis of which revealed that this region contains three phylogenetically conserved, putative hypoxia-response elements (HRE; 5′-NCGTGN-3′) (Xie and Collins, 2011, 2013).

Fibroblasts incubated for 3–10 days and mosaic cultures incubated for 6–8 weeks revealed that a large amount of functional ATP7A is advantagous in high concentrations of copper, whereas a low amount/zero of functional ATP7A is advantageous in low concentrations of copper. Both after 3 and 10 days of treatment, the effect on the transcription level could be visualized, whereas in the mosaic cell culture the effect was seen on clonal selection. The lack of decreased expression of *ATP7A* in WD fibroblasts after BCS treatment might be a result of the absence of functional ATP7B protein in these cells, thereby reducing the demand for a downregulation of *ATP7A*. Furthermore, in both types of experiments we found that cultures grown in basic, control medium also led to an increased expression of *ATP7A* and increased the growth of cells with the wild-type *ATP7A*. An increase in the *ATP7B* transcript level after long-term culturing was only observed for the WD fibroblasts. This might indicate that even under normal conditions, an increased amount of ATP7A is an advantage, whereas an increased amount of ATP7B is an advantage only if the level of functional ATP7B is lower than normal, e.g., as a result of mutations.

A large fraction of the cells died in a mosaic culture with cells from a female carrier grown in medium supplemented with 100–400 μM CuCl_2_ or 10–50 μM BCS, respectively, for either 6 or 8 weeks. This suggests that growth selection is a likely explanation for the observed skewed X-inactivation pattern in these cells. Our results indicate that the two different cell types have complementary growth characteristics. In the presence of a low concentration of copper, the selection went against cells with the active normal X-chromosome, whereas in the presence of a high concentration of copper, the selection went against cells with the active mutant X-chromosome.

Amplification of the *ATP7A* gene has previously been observed in selected CHO cells after stepwise increments of copper in the growth medium (Camakaris et al., 1995). The copper concentration was increased from 142 μM to 205 μM over 27 days, and the cells were cultured for an additional 99 days at the latter concentration. Both mechanisms: *ATP7A* gene amplification, and growth advantage of the cells that express the normal *ATP7A* gene show that the *ATP7A* gene is essential for the cells under conditions with high concentrations of copper. In contrast, the growth advantage of the cells that express the mutant *ATP7A* gene indicates that the absence of a functional *ATP7A* gene is more advantageous if no copper is present. To our knowledge, a positive selection of cells without a functional *ATP7A* gene an effect of the lack of copper has not been reported previously.

Affected carriers of MD are rare. In a total of 517 families we know of 9 confirmed affected females. However, we are aware of the fact that there are mildly affected females who have not been characterized as manifesting carriers. We know of several girls with pili torti, hypopigmentation of the skin or other mild symptoms of MD. The total number of females with symptoms is unknown. It is possible that copper treatment during pregnancy decreases the brain damage in heterozygous female fetuses both indirectly by clonal cell selection of the developing cells that express the normal *ATP7A* gene, and directly by the transport of copper to copper-requiring enzymes. Thus the serum copper level in pregnant heterozygous females should remain within the upper level of the reference interval in order to diminish the symptoms of MD and, in particular, brain damage in both hemi- and heterozygous fetuses. Copper supplements during pregnancy should be considered.

The X-inactivation pattern in a MD mice model should be determined. It would reveal the effect of prenatal copper treatment on the X-inactivation pattern in brain tissue in heterozygous female mouse fetuses.

In X-linked recessive diseases, females are rarely affected, probably due to skewed inactivation of the X-chromosome harboring the mutant allele. Identification of small molecules leading to negative selection against cells deficient in the disease-related gene might be relevant for other X-linked diseases where the phenotype in females could be hampered due to selection of cells with the active normal X-chromosome.

## Materials and Methods

### Chemicals

BCS (Bathocuproinedisulfonic acid disodium salt, Cu+ chelator), DEDTC (Sodium diethyldithiocarbamate trihydrate, copper chelator), CuCl2, Ferrozine (Fe2+ chelator) DTPA (Diethylenetriaminepentaacetic acid, Fe2+ chelator). BCS was from Pierce^1^, all other chemicals, including insulin were from Sigma^2^. The cells were grown in normal medium (see below) supplemented with: BCS (150 μM), CuCl_2_ (150 μM), DEDTC (50 μM), Ferrozine (5 mM), DTPA (10 μM), FeCl3 (150 μM), Insulin (20 ng/ml), 20 ng/ml Insulin + 150 μM BCS, or 20 ng/ml Insulin + 150 μM CuCl_2_.

### Cell Cultures

Skin fibroblasts obtained from the patients and controls were cultured in a 1:1 mixture of RPMI 1640^3^ (ICN Biomedicals, Inc) with 20 mM HEPES and a nutrient mixture of F-10 Ham’s medium, supplemented with 7.5% Amnio Max – C100 supplement^4^ (Life Technologies), 4% fetal calf serum, penicillin, and streptomycin.

Control fibroblast culture, a culture from a MD patient (with a deletion of exon 3–23 of *ATP7A* (c.121-?-8333+?del) and a culture from a WD patient homozygous for the *ATP7B* mutation c.2333G >T, p.(Arg778Leu) was used for the 3–10 day-treatment experiments. The mutations were verified in DNA isolated from blood from the patients.

Two cultures were obtained from unaffected carriers, Carrier 1 (family 9720,3373-84H) and Carrier 2 (family 9720; 3372-84H) and used for 6–8 week-treatment experiments, Carrier 1 and Carrier 2 are both heterozygote for the *ATP7A* mutation p.Gly1315Arg). One culture was obtained from a manifesting carrier, Carrier 3 (family F1, M85-3426H) heterozygote for the *ATP7A* mutation c.1946+5G > A. In addition three control cultures were included:

Control 1 (family 9720, 3540-86H), Control 2 (family F1, M95-24668H), and Control 3 (family F4, M85-2427H). The families have been described previously (Møller et al., 2012).

The fibroblast cultures were cultured at 37^o^C as described previously (Møller et al., 2012). The cells were treated for 3 days, 10 days, 6 or 8 weeks with either BCS or CuCl_2_ and subsequently harvested for RNA or DNA isolation. The RNA was used for RT-PCR analysis and the DNA for X-inactivation studies.

### Quantitation of *ATP7A* and *ATP7B* Transcript

Total RNA was isolated with the Rneasy Mini kit (Qiagen) and single-stranded cDNA was synthesized by reverse transcription using the High-Capacity cDNA Archive Kit (applied Biosystems). For relative quantification of *ATP7A* and *ATP7B* transcripts Real-time PCR was carried out on cDNA with an ABI7300 Genetic Analyzer in accordance with the manufacturer’s instructions (Applied Biosystems, Foster, CA, USA). We used FAM-labeled Taq-Man probes and primers that anneal to the junction between exon 11/exon 12 in *ATP7A*, and a FAM-labeled Taq_Man probe annealing to the junction between exon 13/exon 14 in *ATP7B*, respectively, (Applied Biosystems assay numbers: Hs00921956_m1 and Hs01075297_m1). A FAM-probe and primers to human *GAPDH* were used (Applied Biosystems assay numbers: 4326317E) as an endogenous control.

### X-Inactivation Pattern

DNA was isolated by standard procedures (Grimberg et al., 1989). The methylation status of the AR gene was used as an indicator for X-chromosome inactivation (La Spada et al., 1991; Allen et al., 1992). The amplified products were analyzed in an ABI 3130XL Genetic Analyzer, and the ratio between the signal intensities of the two alleles was calculated using GeneMapper 3.0 software (Applied Biosystems, Foster, CA, USA). The ratio between signals from the two alleles in the undigested sample was used as a correction factor.

### Statistical Methods

Data are presented as mean values ± SD. Statistical analysis was performed with the bilateral Student’s *t*-test using Statgraphics 5.1 (Manugistics, USA). *P* ≤ 0.05, *P* ≤ 0.01, and *P* ≤ 0.001 were considered significant and are denoted with one, two and three asterisks, respectively.

## Ethics Statement

Ethics approvals were obtained from the Danish Bioethics Committees for the Capital Region (KF 01-169/03 and H-1-2011-FSP-31). Written informed consent was obtained from the WD patient after information was given about the project “investigation of copper and iron metabolism”. MD fibroblasts were collected for diagnostic purpose, and since it was not possible to get any contact to the patients or the patients guardians, dispensation for obtaining informed consent was given from the Danish Bioethics Committee for the Capital Region.

## Author Contributions

ML, TJ, and LM, designed the experiments. ML and LM performed the experiments. ML and LM drafted the article MO made **Figure 2**. TM and TJ revised the manuscript critically for its significant intellectual content, and the experiment design.

## Conflict of Interest Statement

The authors declare that the research was conducted in the absence of any commercial or financial relationships that could be construed as a potential conflict of interest.

## Footnotes

1 http://www.piercechemical.com

2 https://www.sigmaaldrich.com/denmark.html

3 http://www.mpbio.com

4 http://www.lifetech.com
